# Audiological benefit, quality of life, and factors associated with functional gain in elderly hearing aid users in a developing country between 2017 and 2020: a pre-post-study

**DOI:** 10.1186/s12877-023-04051-5

**Published:** 2023-06-19

**Authors:** Daniel Peñaranda, Lucia C. Pérez-Herrera, Sergio Moreno-López, Lucia Noguera, Diana Hernández, Daniel Martínez, Augusto Peñaranda

**Affiliations:** 1 Otolaryngology Section, Fundación Universitaria de Ciencias de la Salud, Cra. 19 # 8 A – 32, Bogotá, Colombia; 2 Otolaryngology and Allergy Research groups, Unidad Médico Quirúrgica de Otorrinolaringología (UNIMEQ-ORL), Av. 9 # 116-20, Bogotá, Colombia; 3 School of medicine, Universidad de Los Andes, Cra. 1 Nº 18A – 12, Bogotá, Colombia; 4 Otolaryngology Department, Fundación Santa Fe de Bogotá, Cra. 7 #117 – 15, Bogotá, Colombia; 5 Otolaryngology Department, Universidad Nacional de Colombia, Av. Cra. 30 #45- 03, Bogotá, Colombia

**Keywords:** Hearing aids, Abbreviated Profile of hearing aid, Glasgow Benefit Inventory, Quality-of-life

## Abstract

**Background:**

Sensorineural hearing loss (SNHL) is the most common auditory deficit in older adults and may lead to quality-of-life deterioration. However, few studies have been performed in low/middle-income countries, particularly in Latin America. This study aimed to assess the audiological benefit, quality of life, and factors associated with functional gain in elderly hearing aid users in the Fundación Santa Fe de Bogotá and UNIMEQ-ORL, two otology referral centers in Colombia.

**Design:**

Pre-post study that included hearing aid users at the otology consult of the Fundación Santa Fe de Bogotá and UNIMEQ-ORL between June 2017 and December 2020. Glasgow Benefit Inventory (GBI) and Abbreviated Profile of Hearing Aid Benefit (APHAB) questionnaires were applied. Audiometric (0,5 kHz to 4 kHz) and speech audiometry results were collected.

**Results:**

A total of 75 participants (132 ears) were included. The mean age was 70.73 years (SD: 12.66). The median hearing aid use in years was 0.71 (IQR: 0.64–0.90). Mean change in speech audiometry was − 26.53dB (95%CI: -28.09, -24.97; p < 0.001), in functional gain was − 21.75dB (-23.81, -19.68; p < 0.001). The mean changes in the APHAB domains were Ease of Communication: -37.85 (95%CI: -43.01; -32.7), Background Noise: -3.51 (-6.06; -0.95), and Aversiveness of Sounds: -6.9 (-2.04; 11.77). The GBI assessment of quality of life showed improvement in 100% of the population after the use of hearing aids. The number of years of hearing aids use was associated with functional gain.

**Conclusion:**

The number of years of hearing aids use may impact on the functional gain in these populations. A significant clinical benefit was found in terms of quality of life, communication, and reverberation related to the use of hearing aids. Access to hearing aids should be granted, and public health strategies are needed to grant the access to hearing rehabilitation in these populations.

**Trial registration:**

Fundación Santa Fe de Bogotá (Protocol Number: CCEI-12666-2020).

## Introduction

Hearing loss affects almost 81.4% of adults 80 years or older in the United States [1]. Despite this high prevalence of hearing loss among elderly populations, only 15.5–29% of this population reported access to hearing aid or adequate hearing aid usage [1]. Thus, the prevalence of hearing loss could show variations between different populations, social variables, and access to hearing aids could also be affected by healthcare barriers and environmental factors in developing countries [2, 3]. Early identification of hearing loss followed by an appropriate intervention would reduce its impact on the quality of life [4]. In Bogotá, the capital city of Colombia, Cano et al. reported a prevalence of hearing loss of 46.1% in adults over 70 years or older, of which only 15% had access to hearing aids [5]. Current studies suggest a reduction in the hearing loss burden in high-income countries in North America and Europe due to hearing awareness, screening, and rehabilitation interventions [6]. However, there is still limited data about hearing loss and rehabilitation interventions in low- to middle-income countries [6, 7]. To date, most hearing care strategies in Latin American countries are based on research from high-income North American and European countries [8]. Considering this population bias, the economic differences, and the epidemiologic particularities of low-/middle-income Latin American countries, research studies are needed to develop community-based strategies for hearing loss rehabilitation in these countries.

Hearing impairment has commonly been associated with functional disability, quality of life deterioration, and emotional difficulties [9, 10]. In Colombia, a higher prevalence of hearing loss has been described in illiterate adults and in elderly adults who belonged to lower socioeconomic status [5]. Colombia is a low- to middle-income country with one of the lowest per capita health expenditures ($1.335 United Stated Dollars in 2021) and the highest levels of poverty and income inequality in Latin America [11]. The burden of hearing loss in elderly populations in Colombia may remain high probably due to inadequacies of public health policies, and the inequalities in access to health services [5, 12–14]. This economic scenario is similar to most low-/middle-income Latin American countries [11]. To date, only one study in an elderly Colombian population has described a significant impairment in the quality of life of adults with hearing loss using the EuroQoL-Visual Analogue Scale, EQ-VAS [5]. However, no study has assessed the auditory benefit and changes in the quality of life before and after the adaptation of hearing aid in elderly adults with Sensorineural Hearing Loss (SNHL) in Colombia. There are few studies in low- to middle-income Latin American countries assessing these outcomes. This study aimed to assess the audiological benefit, quality of life, and factors associated with functional gain in hearing aid users with SNHL treated at the Hospital Fundación Santa Fe de Bogotá (FSFB) and UNIMEQ-ORL, Colombia.

## Methods

### Study design

This was an observational, analytical, pre-post study conducted at two otolaryngology referral centers in Bogotá, Colombia: the FSFB and UNIMEQ-ORL, between January 2017 and December 2020. Therefore, this study aimed to assess the possible changes in auditory benefit, quality of life, and factors associated with functional gain in patients with hearing aids. Patients under 70 years old received self-administered written questionnaires, and patients over 70 years old were assessed by their legal tutors to answer the questionnaires. All patients had a clinical diagnosis of sensorineural hearing loss that was supported by audiometric results and was performed by an otolaryngologist and an audiologist. The questionnaires were applied during the audiology appointments by two audiologists trained in the application of these tools. According to the Helsinki Declaration, the ethics committee of the FSFB approved this study (CCEI-12666-2020). Informed consent was obtained from all the participants and/or their legal tutors. No incentives were offered for study participation.

The FSFB and UNIMEQ-ORL are both healthcare centers located in Bogotá, the capital city of Colombia. Patients from all over the country visit these institutions seeking for hearing rehabilitation and otology healthcare. Both institutions treat patients affiliated to private Health Promoting Entities which provide health insurance packages to all socioeconomic-status populations.

### Study population

The sample size was calculated based on an average change in the APHAB score in the pretest of -4.9 units with a standard deviation of 12.2 points, a power of 90% [15], and based on the calculation of the size sample shown below [16]:

$$n=\frac{2{\left({z}_{1-\alpha }+{z}_{1-\beta }\right)}^{2}}{{\delta }^{2}}+\frac{{z}_{1-\frac{\alpha }{2}}^{2}}{2}$$

Where:

$$\delta =\frac{\left|{\mu }_{z}\right|}{{\sigma }_{z}}=\frac{|{\mu }_{x}-{\mu }_{y}|}{\sqrt{({{\sigma }_{x}^{2}+\sigma }_{y}^{2}-2\rho {\sigma }_{x}{\sigma }_{y})} }$$

And with an exposed / unexposed ratio equal to 1 (r = 1), the sample size is equal to 75 subjects. Regarding the sample selection method, a non-probabilistic, consecutive sampling was conducted.

#### Audiometric testing

Audiometry was conducted considering the international standardized protocols to measure the intensity in decibels (dB) at which a tone could be heard by the individuals at a specific frequency [17]. An R37a (Resonance) diagnostic audiometer was used to measure the air and bone conduction thresholds. The diagnostic audiometer calibration protocol was performed considering the American National Standards Institute (ANSI) 3.6 guidelines, 2018 edition [18]. All the audiometric tests were performed following the criteria of ambient noise levels for audiometric test rooms (ANSI/ASA S3.1-1999 (R2013) standard guidelines [18] and were performed in a double-walled soundproof booth (Amplivox). The audiologists used a bone conduction transducer to assess bone conduction, and Radioear DD-45 supra-aural headphones to assess the pure tone audiometry conventional frequencies. Two professional audiologists trained for this study and with wide clinical experience performed the audiometric testing. The audiometric frequencies were assessed through pure tone audiometry for conventional frequencies (0.25- 8 kHz). Tympanometry testing was not performed in the study. Finally, the speech audiometry assessment was performed in quiet in a double-walled soundproof booth (Amplivox) following the “Guidelines for Determining the Threshold Level of Speech” of the American Speech-Language-Hearing Association [19]. The speech reception threshold (SRT) was the lowest decibel level at which the participant correctly repeated 50% of the test words. The word recognition score (WRS) was obtained using phonetically balanced, monosyllabic words usually presented at 30 dB above the hearing threshold obtained from the pure-tone audiogram.

### Questionnaires and tools

The Glasgow Benefit Inventory (GBI) scale was applied to the population study one year after hearing aids indication to assess the quality of life. This scale is sensitive to changes related to otolaryngological interventions. The GBI has 18 questions and 3 domains: 12 questions assess the change in “General benefit”, 3 questions assess “Physical benefit”, and 3 questions are related to the “Social support changes”. It gives a score of -100 (negative benefit), 0 (no benefit), and + 100 (positive benefit) [20]. On the other hand, the Abbreviated Profile of Hearing Aid Benefit (APHAB) is a self-assessment disease-specific disability questionnaire with 24 items divided into four 6-item subscales. The APHAB questionnaire allows patients to measure difficulties with speech or noises in various everyday environments: ease of communication (EC), listening under reverberant conditions (RV), listening in background noise (BN), and the aversiveness of sounds (AV). The responses can be scored on a 7-point scale. Both, the GBI and APHAB questionnaires are widely accepted and internationally validated instruments.

### Statistical analysis

Statistical analysis was performed using Stata 16MP software. The descriptive analysis calculated absolute and relative frequencies for the qualitative variables. Measures of central tendency (mean and median) were estimated for the quantitative variables. Standard deviation (SD) and interquartile range (IQR) were assessed for the dispersion measures. A “change from baseline” measure in audiometry values and APHAB domains was calculated using the differences between the baseline scores obtained before using the hearing aid and the scores after one year. A bivariate and multivariate analysis between pre- and post-measurement data of these scores were analyzed by the paired samples Wilcoxon test. A stratified analysis was performed by age to assess functional benefits for age groups. A GEE analysis using an unstructured correlation matrix was carried out to assess the effect of the functional gain after use and fitting of a hearing aid for one year. This effect was adjusted considering the clinical and demographic variables with clinical relevance or biological plausibility, and those with a P-value ≤ 0.2 in ANOVA/Friedman test. Model assumptions were validated through a linearity test, an estimation of standardized residuals and leverage values, and a comparison between the crude and the adjusted models. Hypothesis testing to determine the level of statistical significance was performed using a 95% confidence interval and a P-value < 0.05.

## Results

A total of 75 individuals were included, with a mean age of 70.73 years (SD: 12.66 years), 54.67% (n = 41) were female, and 84% were aged over 60 years old. The baseline demographic and clinical characteristics of the study population are described in Table 1. The mean hearing aid usage time in years was 0.82 (SD:0.48). The frequency of hearing aid brands was: Widex 58.67%, Phonak 34.67%, Starkey 4%, and RESound/AOR 2.67%, respectively.

**Table 1:** Baseline clinical and sociodemographic characteristics of the study population

Variables	Total participants (n = 75)	
	n	%
Sex: Female/Male	41/34	54.67/45.33
Age in years ^(a)^	70.73 (12.66)	72.42 (65.19–80.03)
Age group		261
40 years old or less	3	4.00
40 to 60 years-old	9	12.00
60 years old or more	63	84.00
Hearing aid usage time in years ^(a)^	0.82 (0.48)	0.71 (0.64–0.90)
Hearing aid brand		
Widex	44	58.67
Phonak	26	34.67
Starkey	3	4.00
RESound/AOR	2	2.67
GBI score post-test ^(b.c)^		
General benefit	42 (33–50)	
Social support	50 (50–67)	
Physical health	17 (17–33)	
Total GBI	39 (17–47)	
Improvement in General benefit ^(d)^	75	100.00
Improvement in Social support ^(d)^	74	98.67
Improvement in Physical health ^(d)^	67	89.33
Improvement in total GBI ^(d)^	75	100.00

a. Values reported in Means (SD) and Medians (IQR)

b. GBI domains

c. Values reported in Medians (IQR: p25-p75)

d. Corresponds to the number of subjects and percentage of the population with improvement classified by each GBI domain

### Changes in audiological outcomes

**Fig. 1:**
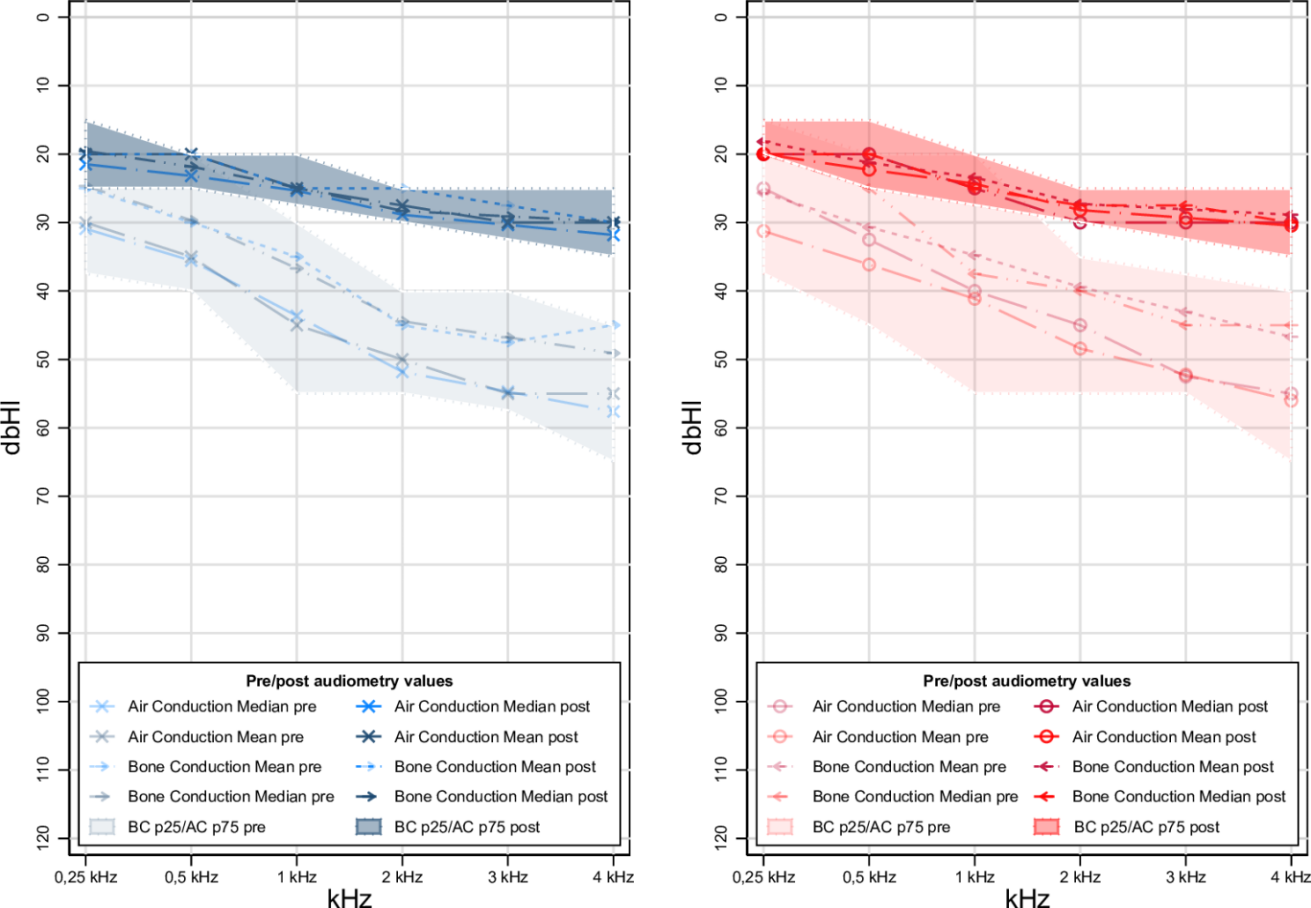
Pre and post audiometric values for right and left ear

In terms of audiometric values, the pre/post and mean changes for the population were stratified by age group (< 60 years vs. > 60 years) and are described in Table 2. Figure 1 shows the pre and post audiometric values of the study population for each ear.All these values were statistically significant. The overall change in the discrimination percentage of the speech audiometry was 4.2% (2.73;5.66), while the mean change in the speechaudiometry values was − 26.53dB (-28.09; -24.97).

**Table 2:** Pre and post APHAB scores,audiometric, and speech audiometric results stratified by age groups

Variables	< 60 years		=>60 years		Overall Mean change (post-pre)	p-value
	Pre-test values	Post-test values	Pre-test values	Post-test values		
	Mean (SD)	Mean (SD)	Mean (SD)	Mean (SD)		
APHAB score						
EC	49.42 (25.20)	19.03 (9.48)	55.29 (20.91)	16.01 (10.13)	-37.85 (-43.01; -32.7)	< 0.0001
RV	51.72 (7.70)	50.64 (10.98)	50.77 (8.25)	50.26 (8.50)	-0.6 (-3.64; 2.44)	0.46
BN	54.68 (6.11)	50.86 (6.92)	51.08 (8.60)	47.63 (8.26)	-3.51 (-6.06; -0.95)	0.004
AV	31.90 (17.42)	38.58 (23.39)	31.87 (17.18)	38.81 (16.95)	-6.9 (-2.04; -11.77)	0.007
Percentage of speech audiometry discrimination	92.50 (9.67)	99.17 (2.82)	96.27 (6.06)	100.00 (0.00)	4.2 (2.73; 5.66)	< 0.0001
dB of speech audiometry	79.58 (6.74)	55.00 (8.60)	80.32 (7.15)	53.41 (8.52)	-26.53 (-28.09; -24.97)	< 0.0001
AC values						
0,25 kHz	32.50 (16.35)	22.71 (7.07)	32.70 (17.27)	20.32 (5.82)	-13.03 (-15.8; -10.26)^a^	< 0.0001
0,5 kHz	38.75 (15.27)	25.00 (6.59)	35.91 (17.29)	22.14 (5.57)	-15.3 (-18.03; -12.57) ^a^	< 0.0001
1 kHz	42.50 (13.67)	27.29 (9.55)	42.34 (16.97)	24.88 (5.91)	-19.05 (-21.55; -16.55) ^a^	< 0.0001
2 kHz	49.38 (11.06)	28.75 (6.95)	50.00 (13.22)	28.49 (5.81)	-22.65 (-24.81; -20.49) ^a^	< 0.0001
3 kHz	52.40 (9.77)	29.79 (6.25)	53.63 (12.83)	29.86 (5.87)	-24.79 (-26.87; -22.72) ^a^	< 0.0001
4 kHz	55.00 (47.50–60.00)	30.00 (25.00–35.00)	55.00 (50.00–65.00)	30.00 (25.00–35.00)	-26.93 (-29.19; -24.68) ^a^	< 0.0001
Functional gain in dB	47.69 (10.32)	28.33 (6.33)	47.83 (13.32)	27.32 (5.30)	-21.75 (-23.81; -19.68)	< 0.0001

a. 67 right ear and 65 left ears analyzed

b. Based on a paired samples Wilcoxon test

### Quality of life

Table 1 shows the results for GBI scores for a total median domain with 39 points (IQR:17–47), median general benefit of 25 points (IQR:33–50), median social support of 50 points (IQR:50–67), and median physical health of 17 points (IQR:17–33), respectively. Thus, 100% of the participants reported improvement in the total GBI score; 100% reported improvement in the general benefit subscale, 98.67% reported improvement in social support, and 89.33% an improvement in physical health (Table 1).

Table 2 shows APHAB scores mean (SD) and median (IQR), discrimination percentage speech audiometry, speech audiometry results, and Air Conduction values audiometry before and after intervention in addition to pre-post differences. These results were stratified by age group (< 60 years vs. > 60 years) to highlight the differences between both groups for each outcome. The crude means differences and confidence intervals of APHAB scores were EC: -37.85 (95%CI: -43.01; -32.7), BN: -3.51 (-6.06; -0.95), and AV: -6.9 (-2.04; -11.77), respectively. These changes in the scores were statistically significant.

### Factors associated with functional gain

Table 3 shows the bivariate and multivariate analyses of the sociodemographic variables for the hearing aids treatment and its effect on audiological benefit. The multivariate and reduced models showed a mean change from baseline in functional gain values of -21.75dB (0.25;0.97) adjusted by sex, age, laterality of the hearing loss, and time of use of hearing aids. These results are consistent with the raw comparisons shown in Table 2 which showed a similar reduction in functional gain in the study population. No collinearity problems were found in the models through the linearity and the goodness-of-fit tests. Overall, these assessments showed good model specifications, and the residual outliers or leverage values did not disturb the models.

**Table 3:** Impact of sex, age, and changes in functional gain assessed trough GEE method

Variable ^(a)^	Functional gain								
	Bivariate model			Multivariate model			Reduced model ^(b)^		
	Coeff.	95% CI		Coeff.	95% CI		Coeff.	95% CI	
Time									
First assessment	0	baseline		0	baseline		0	baseline	
Final assessment	**-21.75**	**-23.68**	**-19.81**	**-21.75**	**-23.68**	**-19.81**	**-21.75**	**-23.69**	**-19.80**
Laterality									
Right ear	0	baseline		0	baseline		--	--	--
Left ear	0.71	-2.87	4.28	0.48	-1.51	2.48	--	--	--
Sex									
Male	0	baseline		0	baseline		0	baseline	
Female	-21.80	-51.11	0.75	-1.57	-4.50	1.36	-1.80	-4.72	1.13
Age	-0.07	-0.18	0.045	-0.06	-0.18	0.050	--	--	--
Time use of hearing aids	-1.01	-3.97	1.95	-1.40	-4.37	1.58	--	--	--
Constant	38.18	36.70	39.66	55.68	47.04	64.32	50.33	47.94	52.71

a. Bolded numbers highlight the significant associations between the variables

b. The reduced model was based on the Furnival−Wilson leaps−and−bounds algorithm. GEE model was estimated with an exchangeable matrix correlation

## Discussion

Despite hearing aids having wide scientific evidence to improve functional disability and quality of life deterioration related to hearing loss in elderly populations, few studies have assessed these benefits in low/middle-income Latin American countries. This study described the audiological benefit and the improvement in patients’ quality of life in older patients with SNHL treated with hearing aids in a low/middle-income Latin American country. Direct costs of SNHL include the use of resources for diagnosis, otology and audiology consults, hearing aids, rehabilitation, and intangible costs such as quality of life deterioration [21–23]. A reduction in the direct costs of SNHL has been reported in hearing aid users from high-income countries [6, 22, 24], but there is no reliable information about these expenses in low/middle-income Latin-American countries. In this study, we describe a significant improvement in terms of quality of life assessed with GBI in 100% of the study population related to hearing aids, which may reduce the burden of hearing loss in this population.

Despite the Colombian health insurance packages include low-cost hearing aids for the population with SNHL, difficulties in access, quality limitations, and lack of follow-up have been described [25, 26]. Considering that Latin American countries have some of the lowest per capita health expenditures [11], this information is essential to design community-based interventions to provide these patients with better care. Moreover, patients in low- and middle-income Latin American countries are exposed to inadequacies of public health policies and inequalities in access to otolaryngology services [5, 12–14]. Therefore, this preliminary evidence supports the need for hearing care campaigns for low-/middle-income countries aiming to improve the diagnosis, access to better treatments, and granting a follow-up rehabilitation process in these populations. Due to the economic and epidemiologic particularities of Latin American countries, cost-benefit research studies addressing the main difficulties in access to hearing aid rehabilitation are needed.

Regarding the audiometric values, an improvement of at least 13 dB was found for the speech frequencies (250–4000 Hz). Similarly, a prior study reported an improvement up to 7 dB +/- 16 dB for the mean pure-tone average at speech frequencies using bone-anchored hearing aids (BAHA) [27]. These findings were also supported by the speech audiometry values that showed significant improvement in the speaking discrimination of words at lower dB values. Moreover, the stratified analysis in Table 2 highlighted the higher functional benefits for the older age groups in this population. Likewise, prior studies in high-income countries have described that hearing aids can provide benefits for both younger and older populations with hearing loss, but the benefits may be more significant in older adults [28–30]. Overall, these results highlight the benefit of hearing rehabilitation for all ages and the higher benefit in older populations.

To gain insight into the quality-of-life changes of this population, the main domains of the GBI score were assessed (general benefit, physical benefit, and social support changes). Overall, the results for GBI scores showed a significant benefit for all the domains of the scale, and up to 100% of the participants reported an improvement in the total GBI score. These findings are similar to a prior study among 134 older patients who received BAHA for SNHL in the Netherlands that reported positive GBI scores in 84% of the population, and a general satisfaction reported by 71% of the population [27] Likewise, a systematic review of randomized controlled trials from high-income countries highlighted that hearing aids improve adults’ quality of life by improving physical, social, emotional and mental well-being [31]. This scenario was similar to our results applying the APHAB questionnaire as the scores showed statistically significant changes in the domains of communication, noise, and aversiveness. We highlight that the higher change was found for the EC domain (-37.85; -43.01, -32.7). Similarly, using the APHAB to assess the changes in quality of life, De Wolf et al. described that among 134 older users of BAHA, 80% of the population reported a significant reduction in their daily problems, and the best scores were obtained for the EC subscale [27]. Our results were similar to these previous findings, and we stand out that hearing aids improve communication with others, which was described as the most important change for the patients.

In terms of the BN (-3.51; -6.06; -0.95), and AV (-6.9; -2.04; -11.77) domains, our population reported less favorable results. Thus, these findings could be explained by differences in hearing aids brands, and access to maintenance of the hearing aids. Prior authors have also reported these differences in elderly hearing aid users [27, 32]. On the other hand, the multivariate and reduced models showed a change of -21.75 dB on functional gain between pre and post audiometric assessments (95% CI: -23.69; -19.80). This scenario could be explained by a longer period of hearing aid use related to advanced handling and higher experience in the maintenance of hearing aids. Hickson et al. described five factors associated with higher success in hearing aids in older adults: greater social support; more difficulties with hearing and communication in everyday life before getting hearing aids; more positive attitudes to hearing aids; greater perceived self-efficacy for advanced handling of hearing aids; or who were receiving more gain from their devices [33].

Overall, these findings highlight the importance of addressing sociodemographic factors to support older adults in achieving success with hearing aids. Finally, we stand out that prior studies have described difficulties in reaching otolaryngology specialists in low/middle-income Latin American countries [34, 35], and additional strategies are needed to grant hearing aid access to older populations in these countries. Access to hearing aids should be granted, and public health strategies are needed to grant the access to hearing rehabilitation in these populations.

Among the strengths of the study, we highlight that the questionnaires were developed by one otolaryngologist and one audiologist with wide clinical experience, and the sociodemographic information was obtained by trained professionals to minimize measurement bias. Moreover, the otolaryngologist of this study reviewed all the information about outcomes and audiological treatment. We highlight that the intensity of audiological change and quality of life were assessed through validated scales in these constructs, so reporter bias and overestimation of the possible benefits in these outcomes were reduced [36]. Moreover, the initial and follow-up audiometric studies were performed in the same audiological center, which would reduce the heterogeneity between different audiometric centers and equipment.

About the limitations of the study, we stand out the cross-sectional design of this study which would also lead to recall bias, considering that most hearing aid patients had to recall their benefit compared to several years ago. However, we also stand out that the most accurate way to evaluate health-related quality of life is to administer the questionnaires before hearing aid fitting and to repeat these measurements after a certain interval which would avoid recall bias [27]. We followed these recommendations and applied the questionnaires before and after hearing aid fitting. Moreover, a higher sample size could improve the generalization of these results. An additional limitation of the study design was the interview style questionnaires. Despite the questionnaires were applied by two audiologists trained in the application of these tools, an interview bias has been described in interview studies [37]. Further studies in these populations are needed, particularly assessing the cost-benefit of hearing aids in developing countries.

## Conclusion

A significant clinical benefit was found in terms of audiological benefit, communication, reverberation, and quality of life probably due to the use of hearing aids.

## Data Availability

Part of the data generated or analyzed during this study is included in this published article. Full datasets generated during and/or analyzed during the current study are available from the corresponding author on reasonable request.

## References

[1] Sharma RK, Lalwani AK, Golub JS. Prevalence and Severity of Hearing Loss in the Older Old Population. JAMA Otolaryngology–Head & Neck Surgery [Internet]. 2020 Aug 1;146(8):762–3. Available from: 10.1001/jamaoto.2020.0900. 10.1001/jamaoto.2020.0900 PMC7317651 32584401

[2] McPherson B. Hearing assistive technologies in developing countries: background, achievements and challenges. Disabil Rehabil Assist Technol [Internet]. 2014 Sep 1;9(5):360–4. Available from: 10.3109/17483107.2014.907365. 10.3109/17483107.2014.907365 24702607

[3] Barillari U. The epidemiology of age-related hearing loss, social aspects and interaction with chronic disease of older adults. BMC Geriatr. 2010 Aug 19;10(S1):L49.

[4] Gao J, Hu H, Yao L. The role of social engagement in the association of self-reported hearing loss and health-related quality of life. BMC Geriatr. 2020 Dec;25(1):182. 10.1186/s12877-020-01581-0 PMC7249415 32450797

[5] Cano CA, Borda MG, Arciniegas AJ, Parra JS. Problemas de la audición en el adulto mayor, factores asociados y calidad de vida: estudio SABE, Bogotá, Colombia. Biomédica [Internet]. 2014 Dec 1;34(4):574–9. Available from: https://revistabiomedica.org/index.php/biomedica/article/view/2352. 10.1590/S0120-41572014000400010 25504246

[6] Eubank TN, Beukes EW, Swanepoel DW, Kemp KG, Manchaiah V. Community-based assessment and rehabilitation of hearing loss: a scoping review. Health Soc Care Community. 2022 Sep;30(5). 10.1111/hsc.13846 35648649

[7] Haile LM, Kamenov K, Briant PS, Orji AU, Steinmetz JD, Abdoli A, et al. Hearing loss prevalence and years lived with disability, 1990–2019: findings from the global burden of Disease Study 2019. The Lancet. 2021 Mar;397(10278):996–1009. 10.1016/S0140-6736(21)00516-X PMC7960691 33714390

[8] Restrepo N, Castillo M. Metodología para la toma de decisiones en el estudio de costo-efectividad de los tratamientos para la sordera neurosensorial bilateral profunda en niños [Internet]. Universidad de Los Andes, editor. Bogota: Universidad de Los Andes; 2008. Available from: https://univdelosandes.on.worldcat.org/search?databaseList=638&queryString=sordera+neurosensorial+bilateral+profunda+en+niños&clusterResults=true#/oclc/915975172

[9] Ciorba A, Bianchini C, Pelucchi S, Pastore A (2012). The impact of hearing loss on the quality of life of elderly adults. Clin Interv Aging.

[10] Cassandro E, Chiarella G. Age-related hearing loss: biological aspects. BMC Geriatr 2010 Aug 27;10(S1):L82.

[11] OECD (2022). OECD economic surveys: Colombia 2022.

[12] Bright T, Mújica OJ, Ramke J, Moreno CM, Der C, Melendez A et al. Inequality in the distribution of ear, nose and throat specialists in 15 latin american countries: an ecological study. BMJ Open 2019 Jul 19;9(7):e030220. 10.1136/bmjopen-2019-030220 PMC6661698 31326937

[13] Bernal R, Cardenas M. Race and Ethnic Inequality in Health and Health Care in Colombia. [Internet]. Bogotá; 2005 [cited 2022 Nov 20]. Report No.: 29. Available from: https://repository.fedesarrollo.org.co/bitstream/handle/11445/811/WP_2005_No_29.pdf?sequence=1&isAllowed=y

[14] Ruano AL, Rodríguez D, Rossi PG, Maceira D. Understanding inequities in health and health systems in Latin America and the Caribbean: a thematic series. Int J Equity Health 2021 Dec 6;20(1):94. 10.1186/s12939-021-01426-1 PMC8023548 33823879

[15] Gil D, Iorio MCM (2010). Formal auditory training in adult hearing aid users. Clinics.

[16] Machin D, Campbell M, Tan S, Tan SH. Sample Size Tables for Clinical Studies, Third Edition. Wiley-Blackwell. 2011. 314 p.

[17] Niskar AS, Kieszak SM, Holmes AE, Esteban E, Rubin C, Brody DJ. Estimated Prevalence of Noise-Induced Hearing Threshold Shifts Among Children 6 to 19 Years of Age: The Third National Health and Nutrition Examination Survey, 1988–1994, United States. Pediatrics. 2001 Jul 1;108(1):40–3. 10.1542/peds.108.1.40 11433052

[18] Acoustical Society of America [ASA]. ANSI/ASA S3.6-2018. https://webstore.ansi.org/Standards/ASA/ansiasas32018.

[19] Guidelines for determining threshold level for speech. ASHA. 1988 Mar;30(3):85–9. 3155355

[20] Sanchez-Cuadrado I, Lassaletta L, Perez-Mora R, Muñoz E, Gavilan J. Reliability and validity of the spanish Glasgow Benefit Inventory after cochlear implant surgery in adults. Eur Arch Otorhinolaryngol. 2015 Feb;272(2):333–6. 10.1007/s00405-013-2844-y 24337876

[21] Ciorba A, Bianchini C, Pelucchi S, Pastore A. The impact of hearing loss on the quality of life of elderly adults. Clin Interv Aging 2012 Jun;159. 10.2147/CIA.S26059 PMC3393360 22791988

[22] Crowson MG, Semenov R, Tucci DL, Niparko K. Quality of Life and Cost-Effectiveness of Cochlear Implants: A Narrative Review. 2017;(Ci):236–58. 10.1159/000481767 29262414

[23] Londoño Trujillo MD, Celis Preciado MScD (2016). MD. CA. Medición de costos indirectos en pacientes colombianos con asma. Revista Colombiana de Neumología.

[24] McDaid D, Park AL, Chadha S. Estimating the global costs of hearing loss. Int J Audiol. 2021 Mar 1;60(3):162–70. 10.1080/14992027.2021.1883197 33590787

[25] Borda MG, Reyes-Ortiz CA, Heredia RA, Castellanos-Perilla N, Ayala Copete AM, Soennesyn H, et al. Association between self-reported hearing impairment, use of a hearing aid and performance of instrumental activities of daily living. Arch Gerontol Geriatr. 2019 Jul;83:101–5. 10.1016/j.archger.2019.04.001 30999124

[26] Ministerio de Salud y Protección Social Colombia. ANALYSIS OF HEARING HEALTH IN COLOMBIA [ANÁLISIS DE SITUACIÓN DE LA SALUD AUDITIVA Y COMUNICATIVA EN COLOMBIA] [Internet]. Bogota. ; 2016 [cited 2023 Apr 16]. Available from: https://www.minsalud.gov.co/sites/rid/Lists/BibliotecaDigital/RIDE/VS/PP/ENT/asis-salud-auditiva-2016.pdf.

[27] de Wolf MJF, Shival MLC, Hol MKS, Mylanus EAM, Cremers CWRJ, Snik AFM. Benefit and Quality of Life in Older Bone-Anchored Hearing Aid Users. Otology & Neurotology [Internet]. 2010 Jul;31(5):766–72. Available from: https://journals.lww.com/00129492-201007000-00010. 10.1097/MAO.0b013e3181e3d740 20581615

[28] Sanders ME, Kant E, Smit AL, Stegeman I. The effect of hearing aids on cognitive function: a systematic review. PLoS One 2021 Dec 31;16(12):e0261207. 10.1371/journal.pone.0261207 PMC8719768 34972121

[29] Wells TS, Nickels LD, Rush SR, Musich SA, Wu L, Bhattarai GR et al. Characteristics and Health Outcomes Associated with hearing loss and hearing Aid Use among older adults. J Aging Health 2020 Aug 16;32(7–8):724–34. 10.1177/0898264319848866 PMC7586498 31092107

[30] Sarant J, Harris D, Busby P, Maruff P, Schembri A, Lemke U, et al. The Effect of hearing Aid Use on Cognition in older adults: can we Delay decline or even improve cognitive function? J Clin Med. 2020 Jan;17(1):254. 10.3390/jcm9010254 PMC7020090 31963547

[31] Ferguson MA, Kitterick PT, Chong LY, Edmondson-Jones M, Barker F, Hoare DJ. Hearing aids for mild to moderate hearing loss in adults. Cochrane Database of Systematic Reviews. 2017 Sep 25;2017(9). 10.1002/14651858.CD012023.pub2 PMC6483809 28944461

[32] Cox RM, Alexander GC. The Abbreviated Profile of Hearing Aid Benefit. Ear Hear [Internet]. 1995 Apr;16(2):176–86. Available from: http://journals.lww.com/00003446-199504000-00005. 10.1097/00003446-199504000-00005 7789669

[33] Hickson L, Meyer C, Lovelock K, Lampert M, Khan A. Factors associated with success with hearing aids in older adults. Int J Audiol [Internet]. 2014 Feb 21;53(sup1):S18–27. Available from: http://www.tandfonline.com/doi/full/10.3109/14992027.2013.860488. 10.3109/14992027.2013.860488 24447233

[34] Bright T, Mújica OJ, Ramke J, Moreno CM, Der C, Melendez A et al. Inequality in the distribution of ear, nose and throat specialists in 15 Latin American countries: an ecological study. BMJ Open [Internet]. 2019 Jul 19;9(7):e030220. Available from: http://bmjopen.bmj.com/content/9/7/e030220.abstract. 10.1136/bmjopen-2019-030220 PMC6661698 31326937

[35] Pérez-Herrera LC, Peñaranda D, Moreno-López S, Otoya-Tono AM, Gutiérrez- Velasco L, García JM et al. Associated factors, health-related quality of life, and reported costs of chronic otitis media in adults at two otologic referral centers in a middle-income country. PLoS One [Internet]. 2021 Dec 31;15(12):e0244797. Available from: 10.1371/journal.pone.0244797. 10.1371/journal.pone.0244797 PMC7775072 33382816

[36] Althubaiti A. Information bias in health research: definition, pitfalls, and adjustment methods. J Multidiscip Healthc 2016 May 4;9:211–7. 10.2147/JMDH.S104807 PMC4862344 27217764

[37] Bergelson I, Tracy C, Takacs E. Best Practices for Reducing Bias in the Interview Process. Curr Urol Rep. 2022 Nov 12;23(11):319–25. 10.1007/s11934-022-01116-7 PMC9553626 36222998

